# Supplementary material for: Prevalence and factors associated with multimorbidity among primary care patients with decreased renal function
Source: PLoS One. 2021 Jan 15;16(1):e0245131. doi: 10.1371/journal.pone.0245131 (PMC7810320; doi:10.1371/journal.pone.0245131)
Supplement: S1 Fig — (DOCX) [file pone.0245131.s001.docx]

**Figure S1 - Bar chart showing numbers of OxREN participants with between 1-8 comorbidities, including CKD, stratified by whether or not participants has CKD (CKD established using the CKD-EPI equation).**
