# Supplementary material for: Prevalence and factors associated with multimorbidity among primary care patients with decreased renal function
Source: PLoS One. 2021 Jan 15;16(1):e0245131. doi: 10.1371/journal.pone.0245131 (PMC7810320; doi:10.1371/journal.pone.0245131)
Supplement: S3 Fig — (DOCX) [file pone.0245131.s003.docx]

**Figure S3 – Comparison of combinations of comorbidities other than CKD in participants with CKD (CKD cohort, n=584) and participants without CKD but have had a test suggesting that they have transiently impaired kidney function (non-CKD cohort, n=277)**
