# Supplementary material for: Prevalence and factors associated with multimorbidity among primary care patients with decreased renal function
Source: PLoS One. 2021 Jan 15;16(1):e0245131. doi: 10.1371/journal.pone.0245131 (PMC7810320; doi:10.1371/journal.pone.0245131)
Supplement: S1 Table — (DOCX) [file pone.0245131.s004.docx]

**Table S1 Compare prevalence of comorbidities in OxRen, those with CKD in OxRen, those with stage 3 CKD in OxRen and RRID (Renal Risk in Derby) study(1), a cohort of 1741 participants with stage 3 CKD.**

| **Condition** | **OxRen Prevalence (%)**  **N=861** | **OxRen CKD only**  **N=584** | **OxRen – Stage 3 only**  **N=379** | **RRID Prevalence (%)**  **N=1741** |
| --- | --- | --- | --- | --- |
| Hypertension | 59.5% | 64.0% | 62.3% | 87.8% |
| Diabetes | 15.5% | 18.7% | 16.9% | 16.9% |
| Ischaemic heart disease | 17.0% | 19.4% | 18.7% | 22.9% |
| Thyroid disorder | 12.3% | 11.5% | 11.9% | 11.9% |
| Cerebrovascular disease | 8.5% | 5.7% | 9.0% | 11.5% |
| Peripheral vascular disease | 3.4% | 3.1% | 2.4% | 4.7% |
| Anaemia | 10.5% | 12.2% | 11.9% | 24.0% |
| Heart failure | 4.5% | 5.5% | 6.1% | 3.5% |

1. Fraser SD, Roderick PJ, May CR, McIntyre N, McIntyre C, Fluck RJ, et al. The burden of comorbidity in people with chronic kidney disease stage 3: a cohort study. BMC nephrology. 2015;16:193.
