# Supplementary material for: Prevalence and factors associated with multimorbidity among primary care patients with decreased renal function
Source: PLoS One. 2021 Jan 15;16(1):e0245131. doi: 10.1371/journal.pone.0245131 (PMC7810320; doi:10.1371/journal.pone.0245131)
Supplement: S2 Table — (DOCX) [file pone.0245131.s005.docx]

**Table S2 –Prevalence of multimorbidity in people with CKD in the OxREN cohort stratified by whether their CKD was existing or newly diagnosed.**

| **Number of comorbidities** | **Existing CKD**  **N=327** | | **Newly-diagnosed CKD**  **N=257** | |
| --- | --- | --- | --- | --- |
|  | **N** | **% (95%CI)** | **N** | **% (95%CI)** |
| 0 | 0 | - | 0 | - |
| 1 | 35 | 11% (7-14) | 43 | 17% (12-22) |
| 2 | 83 | 25% (21-30) | 71 | 28% (22-33) |
| 3 | 88 | 27% (22-32) | 56 | 22% (17-27) |
| 4 | 60 | 18% (14-23) | 40 | 16% (11-20) |
| 5 or more | 61 | 19% (14-23) | 47 | 18% (14-23) |
| Overall prevalence of multimorbidity | 292 | 89% (86-93) | 214 | 83% (79-88) |
