# Supplementary material for: Prevalence and factors associated with multimorbidity among primary care patients with decreased renal function
Source: PLoS One. 2021 Jan 15;16(1):e0245131. doi: 10.1371/journal.pone.0245131 (PMC7810320; doi:10.1371/journal.pone.0245131)
Supplement: S3 Table — (DOCX) [file pone.0245131.s006.docx]

**Table S3 – Logistic regression to identify predictors of multimorbidity, including stage of CKD and waist circumference and hip circumference instead or waist-to-hip ratio in the analysis**

| **Covariate** | **Univariable analysis, OR and 95% CI** | **P-value** | **Multivariable analysis, OR and 95% CI** | **P-value** |
| --- | --- | --- | --- | --- |
| Age (years) | 1.048 (1.024 to 1.073) | <0.0001 | 1.069 (1.040 to 1.099) | <0.0001 |
| Women | 1.00 (reference) |  | 1.00 (reference) |  |
| Men | 1.206 (0.888 to 1.640) | 0.231 | 0.654 (0.404 to 1.059) | 0.084 |
| BMI (kg/m^2^) | 1.142 (1.101 to 1.184) | <0.0001 | 1.161 (1.079 to 1.250) | <0.0001 |
| Smoking status (compared to never smoker) |  |  |  |  |
| - Never smoker | 1.00 (reference) |  | 1.00 (reference) |  |
| - Current smoker | 1.352 (0.623 to 2.930) | 0.445 | 1.609 (0.689 to 3.756) | 0.271 |
| - Former smoker | 1.495 (1.086 to 2.059) | 0.014 | 1.322 (0.927 to 1.886) | 0.123 |
| Alcohol dose (g/day) | 1.003 (0.988 to 1.019) | 0.680 | 1.009 (0.990 to 1.028) | 0.357 |
| Secondary education | 1.00 (reference) |  | 1.00 (reference) |  |
| Higher education | 0.756 (0.534 to 1.069) | 0.114 | 1.041 (0.705 to 1.536) | 0.840 |
| Stage of CKD |  |  |  |  |
| - No CKD | 1.00 (reference) |  | 1.00 (reference) |  |
| - Stage 1 | 1.463 (0.655 to 3.269) | 0.354 | 2.141 (0.793 to 5.780) | 0.133 |
| - Stage 2 | 1.258 (0.789 to 2.005) | 0.334 | 1.269 (0.760 to 2.117) | 0.362 |
| - Stage 3a | 1.633 (1.108 to 2.407) | 0.013 | 1.174 (0.665 to 2.072) | 0.580 |
| - Stage 3b | 2.519 (1.501 to 4.226) | <0.0001 | 1.346 (0.530 to 3.416) | 0.532 |
| - Stage 4 | 3.884 (1.777 to 8.488) | 0.001 | 1.690 (0.462 to 6.178) | 0.428 |
| Systolic blood pressure (mg Hg) | 1.002 (0.994 to 1.010) | 0.591 | 0.996 (0.984 to 1.008) | 0.489 |
| Diastolic blood pressure (mm Hg) | 0.996 (0.982 to 1.009) | 0.523 | 0.996 (0.976 to 1.015) | 0.672 |
| White ethnicity | 1.00 (reference) |  | 1.00 (reference) |  |
| Non-white ethnicity | 0.632 (0.209 to 1.905) | 0.415 | 0.915 (0.271 to 3.091) | 0.887 |
| Waist circumference (cm) | 1.042 (1.030 to 1.055) | <0.0001 | 1.016 (0.989 to 1.044) | 0.247 |
| Hip circumference (cm) | 1.053 (1.036 to 1.070) | <0.0001 | 0.980 (0.950 to 1.012) | 0.217 |
| eGFR | 0.979 (0.971 to 0.988) | <0.0001 | 0.990 (0.968 to 1.012) | 0.360 |
| UTI | 0.728 (0.536 to 0.989) | 0.042 | 0.757 (0.528 to 1.085) | 0.129 |
