# Supplementary material for: Prevalence and factors associated with multimorbidity among primary care patients with decreased renal function
Source: PLoS One. 2021 Jan 15;16(1):e0245131. doi: 10.1371/journal.pone.0245131 (PMC7810320; doi:10.1371/journal.pone.0245131)
Supplement: Attachment — Submitted filename: Response to reviewers comments.docx [file pone.0245131.s007.docx]

**Plos One – multimorbidity paper - response to reviewer’s comments**

| Reviewer #1: |  |
| --- | --- |
| I congratulate the authors for such a nice study. It's important to delineate the burden of CKD and multi-morbidity at primary care level. I have few suggestions | Thank you for your comments |
| 1. The presentation of results could be sub-headings for better reading. For example if the results are presented as Overall cohort, CKD, Non CKD and Male and female. I found that there was lot of information, especially % all over the place and I moved backwards and forwards to read. | We have restructured the results to findings in the full cohort from the CKD and non-CKD populations.  We have also included another subheading: “*Differences by GFR-estimating equation and sex*” to make comparisons between the MDRD and CKD-EPI equations and males and females. |
| 2. This data from primary care is very important and actually define a more milder cohort of CKD. If you take an average 74 year old white (98%) UK resident with hypertension, obesity and CKD as the top co-morbidities with rest of the conditions at much lower percentages including diabetes, cardiac and other vascular conditions, as seen in your data, this group represents a milder form of disease compared to any group of CKD patients seen in speciality renal practices. For example in out cohort in Australia ( 90% Caucasians) seen in specialist clinics, Obesity is >50%, Diabetes is 44%, hypertension 91%, coronary artery disease 25% and other vascular complications in the range of 10%. (<https://doi.org/10.1111/nep.13567>). Similar is the case in other CKD registry reports. The implications of these comparisons with other international cohorts are huge. Either you evaluated a milder form of disease or your primary care programs are working very well so much so the CKD patients are managed very well at primary care level and mulitmorbidity profile is much contained and better. I suggest that you compare your data locally with national datasets as well as international registry reports like Australian, Japanese, German or European CKD data. I think your data send a much powerful message regarding the success of primary care in your country. | Thank you for your comments.  We have changed the discussion to include a comparisons with literature, first in the UK followed by international settings.  This section in the Discussion has now been substantially revised. |
| Reviewer #2: |  |
| Thank you for the opportunity to review this paper. There are methodological issues which I believe should be addressed.. |  |
| One such issue you describe in your limitations (your population is not representative of the UK population and findings cannot therefore be extrapolated to the general population). | The reviewer has pointed out a concern about the generalisability. We have compared our results with those from other studies and suggested reasons for differences in prevalence of multimorbidity and individual comorbidities. We have included a sentence to explain that we have compared with other studies: *“To be transparent about the generalisability, we have discussed the results in the context of other studies, both in the UK and internationally.”* |
| You must describe laboratory methodology ie serum creatinine assay and its traceability to IDMS and your methodology for detecting a raised ACR of > 3mg/mmol. | We have clarified these points in the text: “*Non-fasting blood and urine samples collected at the baseline study visit were analysed within 24 hours across two laboratories using identical albumin-creatinine ratio (ACR) and isotope dilution mass spectrometry (IDMS)-traceable enzymatic creatinine assays, and reported using the Modification of Diet in Renal Disease (MDRD) eGFR. Both samples for each patient were processed in the same laboratory. The remaining 277 (32.2%) participants did not have a CKD diagnosis, but had one test with either a decreased eGFR (<60 ml/min/1.73m^2^) or a raised non-fasting urinary ACR (>3 mg/mmol), suggesting transiently impaired renal function, or may potentially arise from variability in analytical methods in those near diagnostic thresholds*.” |
| You must also describe your sampling methodology - when were blood samples taken for serum creatinine analysis, were they fasted or non-fasted (relationship to a protein containing meal); when were urine samples for ACR testing taken; were samples stored before analysis. | Please see above |
| You have not defined transiently impaired kidney function nor have you indicated baseline measures or the magnitude of the transient impairment. | The participants we classified as having transiently impaired renal function had a single eGFR or ACR test over the diagnostic threshold, but did not meet the KDIGO criteria for sustained impairment. We have included these participants in our cohort as we believe that they may be more likely to progress to CKD than the general population. We have removed most references to transiently impaired renal function in the text and instead refer to this population as non-CKD  Details of this population, as well as other characteristics of the participants are given in a paper published earlier this year. Rather than repeat these, we have referred the reader to the paper: *“More details of the population included in this analysis are given in our paper summarising the characteristics from the baseline study visit.(28)”* |
| Although correct to say that any test has both physiological and analytical variability without detailing the conditions under which the test sample is taken, the assay used or the magnitude of the abnormal result it is impossible to draw meaningful conclusions | We hope the more detailed methods given above have served to clarify this. |
|  |  |
